# An improved automated diatom detection method based on YOLOv5 framework and its preliminary study for taxonomy recognition in the forensic diatom test

**DOI:** 10.3389/fmicb.2022.963059

**Published:** 2022-08-19

**Authors:** Weimin Yu, Qingqing Xiang, Yingchao Hu, Yukun Du, Xiaodong Kang, Dongyun Zheng, He Shi, Quyi Xu, Zhigang Li, Yong Niu, Chao Liu, Jian Zhao

**Affiliations:** ^1^Jiangsu JITRI Sioux Technologies Co., Ltd., Suzhou, China; ^2^School of Forensic Medicine, Kunming Medical University, Kunming, China; ^3^LabWorld (Suzhou) Intelligent Technology Co., Ltd., Suzhou, China; ^4^School of Forensic Medicine, Southern Medical University, Guangzhou, China; ^5^Key Laboratory of Forensic Pathology, Guangzhou Forensic Science Institute, Ministry of Public Security, Guangzhou, China; ^6^Section of Forensic Sciences, Department of Criminal Investigation, Ministry of Public Security, Beijing, China

**Keywords:** forensic science, drowning, diatom test, artificial intelligence, YOLOv5 framework, microwave digestion-vacuum filtration-automated scanning electron microscopy

## Abstract

The diatom test is a forensic technique that can provide supportive evidence in the diagnosis of drowning but requires the laborious observation and counting of diatoms using a microscopy with too much effort, and therefore it is promising to introduce artificial intelligence (AI) to make the test process automatic. In this article, we propose an artificial intelligence solution based on the YOLOv5 framework for the automatic detection and recognition of the diatom genera. To evaluate the performance of this AI solution in different scenarios, we collected five lab-grown diatom genera and samples of some organic tissues from drowning cases to investigate the potential upper/lower limits of the capability in detecting the diatoms and recognizing their genera. Based on the study of the article, a recall score of 0.95 together with the corresponding precision score of 0.9 were achieved on the samples of the five lab-grown diatom genera *via* cross-validation, and the accuracy of the evaluation in the cases of kidney and liver is above 0.85 based on the precision and recall scores, which demonstrate the effectiveness of the AI solution to be used in drowning forensic routine.

## Introduction

In forensic sciences, it has been widely proved that the diatom test is an effective method for the diagnosis of drowning from other causes of death (Pollanen et al., 1997; Ludes et al., 1999; Zhao et al., 2017). As one of the unicellular algae, the diatoms exist in almost all water bodies, and naturally, they would go along with the inhaled water into the lung of a drowning person, and these diatoms would appear in some other organs like the liver and kidney through the circulation of blood. However, a dead victim that was caused by other reasons but found in a water body would notpass the diatom test on his/her liver and kidney samples due to the end of the blood circulation (Kaushik et al., 2017). Even in drowning cases, there is only a small amount of diatoms in the closed organs which makes it difficult for forensic pathologists to detect them. In addition, there are hundreds of diatom genera living in the world, and the number of the dominant genera in a specific water region is countable, which allows for the construction of a diatom database to infer the drowning site of a drowned body (Zhang et al., 2021).

Either the diagnosis of drowning or the drowning site inference can resort to the diatom test by detecting the diatoms from the sediments in the tissue samples of multiple organs and then identifying their types for statistical analysis. To capture the diatoms varying from a few micrometers to a submillimeter, microscopy is required to scan the images at a magnification from a hundred to a thousand depending on optical microscopy or scanning electron microscopy (SEM). Traditionally, the diatom test always involves large numbers of laborious and tedious observation and search jobs on the scanned optical or SEM images, which have to be physically done by forensic pathologists. This situation is not friendly for practice and is apt to cause high false negative/positive rates due to fatigue and decreased concentration. It is of particular interest for academic research to explore the capability of automatically detecting the diatoms and/or recognizing the genera of the diatoms on optical microscope images (Bueno et al., 2018; Zhou et al., 2019, 2020; Kloster et al., 2020; Krause et al., 2020) or the SEM images (Deng et al., 2020; Yu et al., 2021). These studies are inspired by the development of artificial intelligence recently and especially the giant success of deep learning (LeCun et al., 2015) in image processing and analysis, such as image classification, object detection, and region-of-interest (ROI) segmentation, which then makes it possible to build our own intelligent diatom test solution.

Deep learning is a category of machine learning (Jordan and Mitchell, 2015) that is within the scope of artificial intelligence, and artificial intelligence allows machines to work efficiently and solve problems automatically based on the technologies of machine learning and pattern recognition which is another domain. For machine learning, there is a long history of development and prosperity, and conventionally the machine learning methods always contain a key step called feature engineering to design high-dimensional hand-crafted descriptors for downstream tasks like classification. In Safavian and Landgrebe (1991), Fischer and Bunke (2001), Jalba et al. (2001), and Gloria et al. (2017), a few studies were conducted on the taxonomy of the diatoms on the microscopic images based on machine learning. Various features were proposed to effectively distinguish the diatoms from other objects and these features were generally computed from statistical, textural, and morphological information. Then, a classifier such as a decision tree was trained on the feature data extracted from the given training images to infer the genera of the diatoms. However, conventional machine learning is not very suitable for the detection of diatoms due to the difficulty in encoding the position of diatoms to a high-dimensional feature representation. In Paul and Jones (2001), this challenging work was first and preliminarily evaluated with different visual descriptors and classifiers based on the Viola-Jones object detection framework.

In general, optical microscopy is not that powerful for zooming in on the features of diatoms when compared to scanning electron microscopy; however, the former with the advantage of much lower cost is enough for the classification of the images about if they contain the diatoms or not. In Zhou et al. (2020), 58 sample slides were scanned by a Leica scanner at a 40× magnification, and each slide image was split into a group of 255 × 255 non-overlapping small patches. The deep learning classification model Inception-v3 proposed by Google (Szegedy et al., 2016) was learned on the given training patches for binarily predicting if one test patch includes at least one diatom or not. By sliding window, the location of the diatoms can be coarsely determined. Similarly, the study of taxonomically identifying the morphologically diverse microalgal group of diatoms was reported on Kloster et al. (2020). The images for the study were acquired by an optical scanner with a pixel resolution of 0.1 μm. The classical model VGG16 (Karen and Zisserman, 2014) was adapted for the evaluation and a high F1 score of 0.97 was achieved.

Object detection is not well tackled until the introduction of deep learning on this diatom image processing task (Deng et al., 2009). In this pioneering work, feature engineering is replaced by a deep neural network called R-CNN to automatically learn the representation of a high-dimensional latent space on a large-scale image database ImageNet (Girshick et al., 2014), including 14 million images with rich morphological and textural features, and thus it provides the potential to build a strong capability of generalization. Faster R-CNN (Ren et al., 2017), as the third generation of the R-CNN, is a robust object detection framework that has been used for the detection of the diatoms on the SEM images (Deng et al., 2020) for the diatom test. They compared the results achieved by the faster R-CNN model and three conventional machine learning methods which demonstrated the superiority of deep learning. This is a preliminary investigation on the automatic diatom detection issue, while some detailed information like the magnification of image acquisition and the false negative/positive rates are not mentioned. In Yu et al. (2021), we assessed the performance of detecting the diatoms on an 800× image set and a 1,500× image set with another well-known object detection model RetinaNet (Lin et al., 2017). Both image sets were scanned by a Phenom XL desktop scanning electron microscopy, and we set the magnification to a low-medium level to substantially save the time of scanning which is routinely quite needed. Consequently, a 12% false negative rate and a corresponding 18% false positive rate were achieved. In Krause et al. (2020), the evaluation was performed on a group of two-channel (fluorescence and phase contrast) microscopic images, and the F1 score of 0.82 was achieved on another 600 test images.

In our previous work (Yu et al., 2021), we adapted the deep learning object detection framework RetinaNet for a preliminary evaluation of the SEM-based diatom detection. In Yu et al. (2021), considering the quantity limit of the collected SEM images, we applied a strategy of data augmentation by randomly cropping a single 1,024 × 1,024 SEM image to a local 512 × 512 region that contains at least one diatom for training, and splitting one test image to four 512 × 512 image patches for inference. In this study, it is not necessary because we collected much more images for training and testing. We adopt another AI-based object detection framework YOLOv5 (YOLOv5 GitHub Repository)^1^ which is the latest version of the deep learning architectures YOLO (Redmon et al., 2016). One prominent difference is that the RetinaNet-101 model has more than 5.532 × 10^7^ parameters while a medium YOLOv5 model is more compact with only about 2.104 × 10^7^ parameters, which means there is less computation and faster. In the meanwhile, the YOLOv5 has been proved to be superior to the RetinaNet model for accuracy.

**Figure 1:**
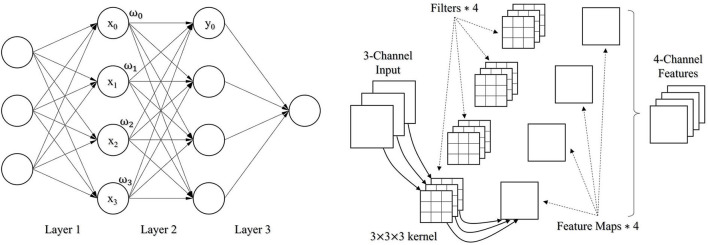
A fully connected network with 3 FC layers (Left); An illustration of how a convolutional layer works (Right).

No matter the RetinaNet or the YOLOv5 object detection method, both have a structure of convolution neural network (CNN) belonging to the scope of deep neural network. A conventional neural network, that is, a fully connected (FC) network is completely based on the connection of adjacent neurons along the direction of propagation (Figure 1, Left). The mathematical form of a fully connected network can be represented as $y=𝟋\left(\overset{N}{\underset{i=0}{\sum }}\omega_{i}\cdot x_{i}+b\right)$, where {ω_*i*_} are the learnable weight parameters, b is either a constant value or a learnable parameter as a bias factor, and F is an activation function like sigmoid or softmax function to involve nonlinearity in the network. In practice, the fully connected network has some issues with handling the tasks like image classification, detection, and segmentation. Particularly, when a fully connected network is a little deep, it is prone to overfitting due to too intensive computation. By contrast, the convolution neural network is the combination of multiple types of neural computing layers, including the convolutional layer, pooling layer, and the mentioned fully connected layer. A convolutional layer convolves an input and passes its result to the next layer. The input is filtered by a set of convolution kernels with a limited number of learnable parameters (Figure 1, Right) compared to the fully connected layer. Therefore, the adaptation of the convolutional layers allows building a deep neural network with a better capability of the fitting. Besides, the pooling layer is used to downsample a feature map by voting on a local, for example, 2 × 2 feature. There are some pooling methods, such as average pooling and max pooling.

Following the previous evaluation (Yu et al., 2021), we continue the work by investigating new deep learning technologies to achieve better performance and developing a practical SEM-based diatom detection technology with an artificial intelligence engine. Moreover, we conducted a more comprehensive study trying to approach the potential upper and lower limits of our proposed method which will be introduced in the following section.

## Materials and methods

**Figure 2:**
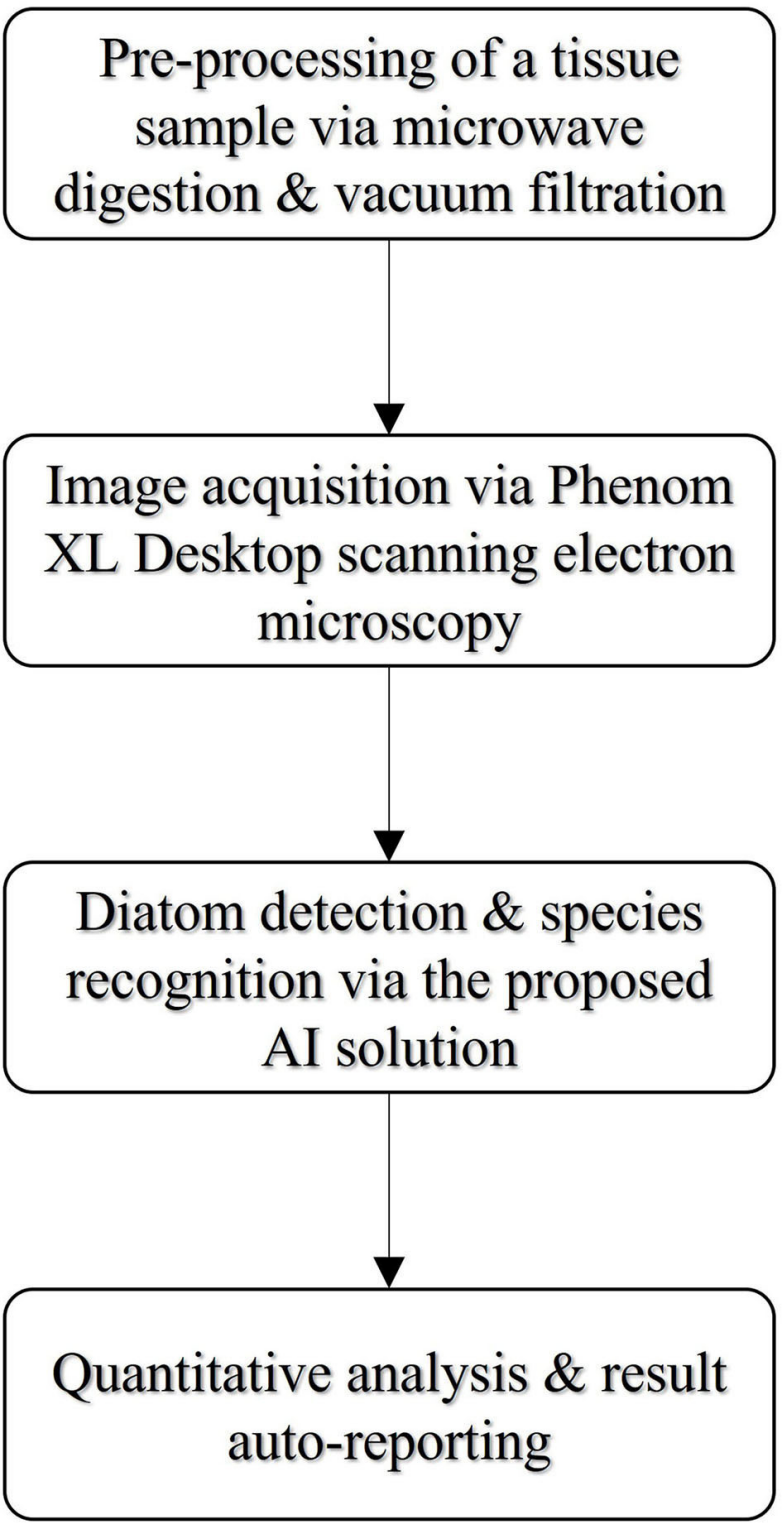
The workflow of the proposed diatom detection and recognition solution.

Figure 2 illustrates the workflow of our proposed SEM-based diatom detection and recognition solution which can be broken down into multiple modules. The workflow begins with a hierarchical pre-processing module combining microwave digestion and vacuum filtration (Zhao et al., 2013, 2017) developed by Guangzhou forensic science institute, and followed by the image acquisition using a Phenom XL desktop SEM at a certain magnification. This workflow has been proved to be a sensitive method for the forensic diatom test (Zhao et al., 2013) compared to the conventional acid digestion method. We scanned the pre-processed tissue samples using back-scatter electron mode (BSE) as images and fed them into our developed detection and recognition AI solution, which is composed of a bunch of automatic functions like the AI-based diatom detection and recognition, quantitative analysis and report generation, and the function of training your own models for some specific sample cases from end-users.

### Data acquisition

To comprehensively evaluate the accuracy of the diatom detection and genus recognition models, we collected three types of sample data as follows:

A. Samples of five lab-grown diatom genera with the names of *Coscinodiscus, Cymbella, Navicula, Nitzschia, and Synedra*, respectively;
B. Samples collected from lung tissues;
C. Samples collected from liver and kidney tissues.

The samples of *Coscinodiscus, Cymbella, Navicula, Nitzschia*, and *Synedra* were provided by the Institute of Hydrobiology, Chinese Academy of Sciences (Volume: 13–15 ml, Concentration: >10^6^, Culture Condition: 25°C). These samples were processed by the Microwave Digestion-Vacuum Filtration-Automated Scanning Electron Microscopy as a Sensitive Method (Zhao et al., 2013).

The SEM membrane samples of lung, liver, and kidney tissues from nine cases that have been involved here are confirmed drowning by the eyewitness and autopsy findings of drowning signs and the exclusion of other injuries, drug, intoxication, alcohol, and medication-related. In this study, we assess the performance of the trained AI models on the given liver and kidney samples *via* cross-validation and demonstrate the efficiency of the solution in the general cases of drowning forensic diatom test. On the other hand, the samples extracted from lung tissue contain various impurities. Although the pre-processing steps of microwave digestion followed by vacuum filtration are applied in our workflow to remove those impurities, there are still many remaining impurities. Therefore, it is a real challenge to well detect and recognize the diatoms located in the SEM images of the lung samples, and herewith, we test the lung samples collected from those drowning cases for the evaluation on some extreme conditions with numerous different sediments which make the background of the images very complicated.

In addition, the samples of the five lab-grown diatom genera were collected from a laboratory environment, and an apparent difference between these samples and the samples collected from the lung, liver, and kidney tissues of drowning corpses is that the acquired SEM images from the lab-grown diatoms suffer less from the interference of impurities. Thus, the given samples are quite appropriate for the quantitative analysis of the potential upper limit performance on both the diatom detection and the genus classification.

The diatom test method that combines microwave digestion (MD) and vacuum filtration (VF) was proposed to replace the conventional pre-processing method based on acid digestion and centrifugation, and the former has a higher time efficiency and a better filtration quality (Zhao et al., 2013). We acquired the SEM images on these processed samples using a Phenom XL desktop SEM at the magnification of 1,500× with a pixel resolution of 0.33 μm and a field of view (FOV) of 336 μm. Each scanned image has a unified size of 1,024 × 1,024 pixels, and the positions and genera labeling of the diatoms were done by two senior forensic pathologists experienced in diatom tests.

For the samples of each lab-grown diatom genera, there are around 2,000 images scanned, and not all of them contain the diatoms (about 46%). Table 1 is the summary of the scanned SEM images of the standard samples evaluated in our study.

**Table 1:** Summary of the SEM images scanned from the standard samples.

**Genera**	**Scanned images**	**Images with diatoms**	**Diatom count**
*Coscinodiscus*	2,018	630	812
*Cymbella*	2,084	672	921
*Navicula*	1,966	930	1,356
*Nitzschia*	1,999	1,476	6,515
*Synedra*	1,875	1,622	5,741
Total	9,942	5,330	15,345

For the lung tissue samples, we mixed all the scanned images for training a robust diatom detection AI model based on a large dataset. In detail, there are totally 2,343 images while 1,783 images contain at least one diatom, and the total number of all the diatoms is 5,899. In addition, there are totally 11 samples collected from the liver and kidney tissues which are described in Table 2. Note that we inherited two sets of images from our previous work (Yu et al., 2021). The first set was scanned at the magnification of 800× (#01) and the second one was acquired under the setting of 1,500× magnification (#02). Besides, there are images from liver samples (#03 → #05) and kidney samples (#06 → #11) randomly selected from the nine drowning cases.

**Table 2:** Summary of the SEM images scanned from the liver and kidney samples.

**Image set**	**Images with diatoms**	**Diatom count**
#01	904	2,789
#02	938	1,168
#03	8	8
#04	509	597
#05	108	113
#06	3	3
#07	1,687	2,125
#08	54	56
#09	69	72
#10	58	60
#11	35	39
Total	4,373	7,030

**Figure 3:**
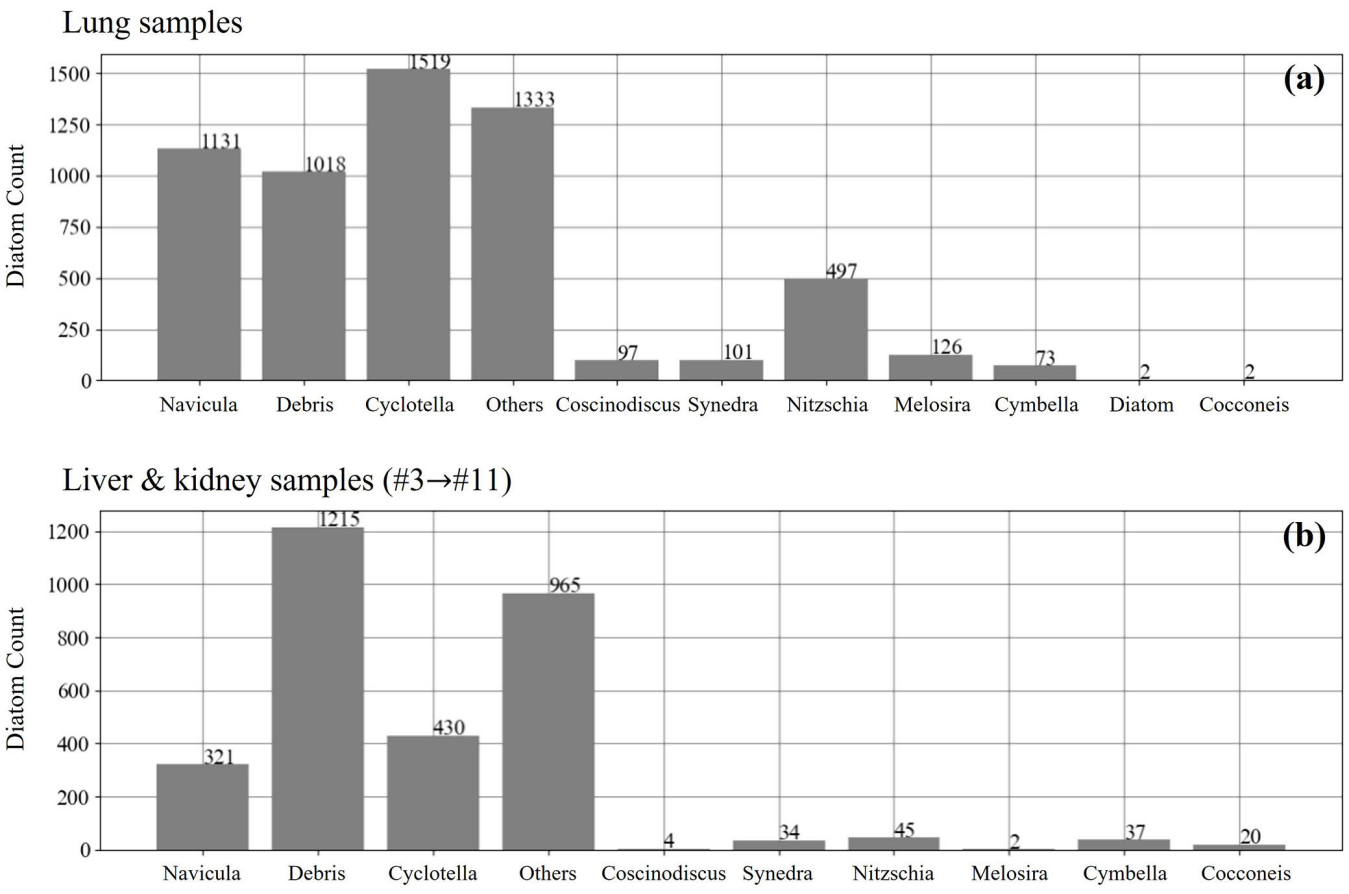
The number distribution of different diatom genus in the lung **(a)**, liver and kidney samples **(b)**.

In comparison to the standard samples, the samples of the lung, liver, and kidney tissues are extracted from the real cases, and the number distribution of different diatom genera is not uniformly distributed for the training of a multi-class recognition AI model that can work well on the inference of all the target genera. The label information of both the lung data and the liver and kidney data is illustrated in Figure 3, and we notice that there are two interference labels named “debris” and “other.” The label “debris” means the incomplete diatoms and the second label “other” denotes those uncommon diatom genera in forensic practice. Therefore, we only conduct the study of assessing the performance of the diatom detection based on the current samples.

### YOLOv5

**Figure 4:**
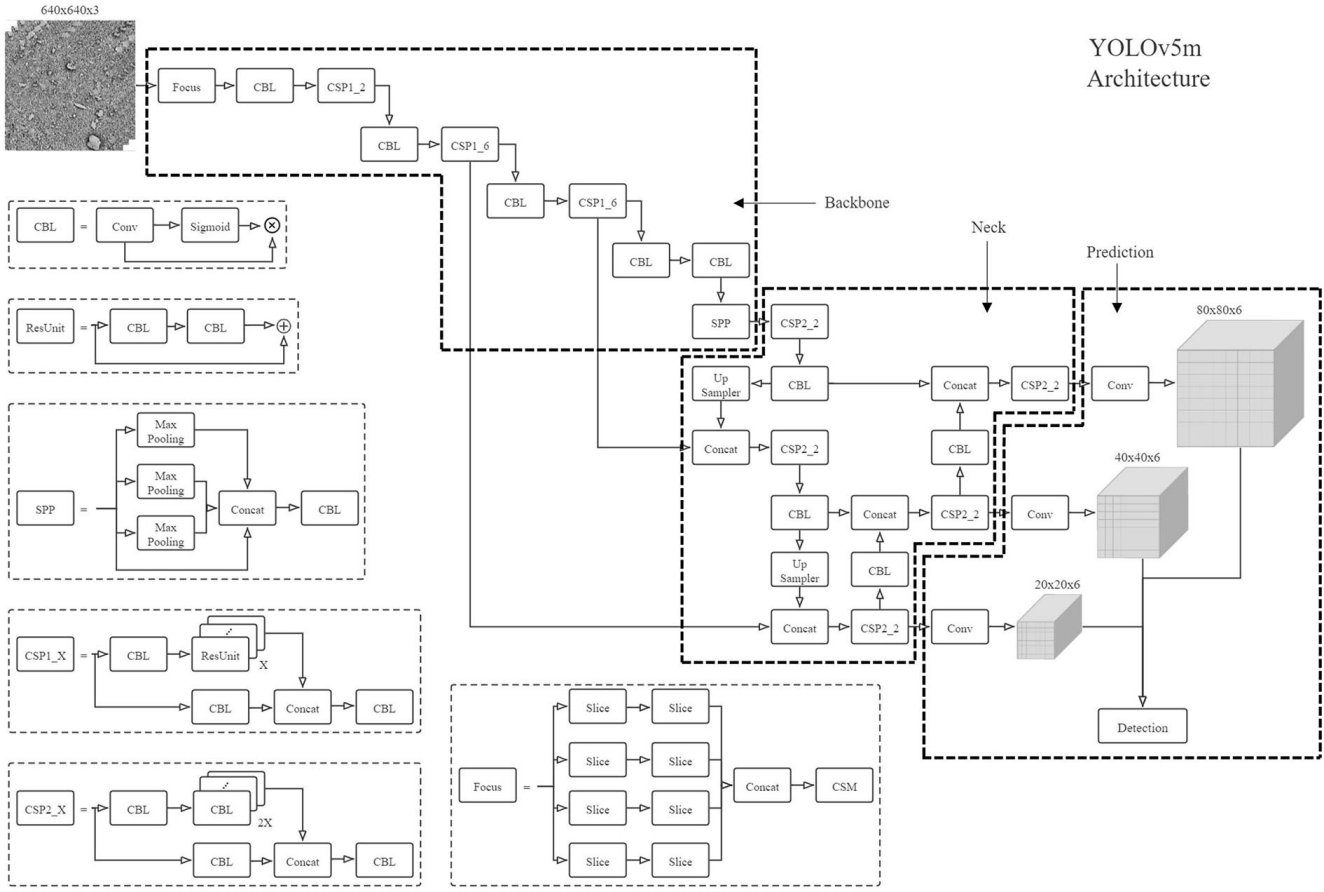
The YOLOv5m architecture.

Same as the RetinaNet framework (Lin et al., 2017), the YOLOv5 is also a one-stage object detection framework that takes a batch of the resized 3-channel SEM images as input and can directly predict the location of the diatoms and optionally their genera. The localization of each diatom candidate is predicted by a sub-regression model as a part of the YOLOv5 detection solution. On the other hand, it has a sub-classification model trained to recognize if the candidate is a real diatom with a confidence prediction. More specifically, the YOLOv5 has four model structures depending on the number of the model layers and parameters, ranging from small to super large, and we picked a medium model YOLOv5m for training and testing to evaluate the performance on the given image data. The YOLOv5m model has the network architecture illustrated in Figure 4, and the architecture can be broken down into a backbone network followed by a neck structure connecting to the section for detection prediction. In detail, the construction of the backbone network is based on the Focus module and CSP module (see Figure 4), and the neck structure is the enhancement of the FPN structure (Lin et al., 2017) appeared in the RetinaNet by adding a structure called PAN for bottom-up path aggregation. The combination of the FPN structure and the PAN structure was originally proposed for image segmentation (Liu et al., 2018) to shorten the information path between lower and topmost features. It was first introduced into YOLOv4 and then in the YOLOv5 framework, this structure was slightly modified with the replacement of some CBL modules by the CSP modules which are constructed based on CBL.

The prediction from the neck structure consists of three outputs with different feature sizes (e.g., 80). The prediction from the neck structure consists of three outputs with different feature sizes (e.g., 80 × 80, 40 × 40, and 20 × 20) and receptive fields. The 3rd dimension of every output is composed of four coordinates of a bounding box, one confidence score, and *K* probability values for the genus recognition (Redmon et al., 2016), and *K* = 1 if we only consider the diatom detection for counting the diatoms without the genus recognition. In addition, corresponding to the diatom classification and the diatom localization regression, the loss function used for the YOLOv5m model training can be divided into classification loss and bounding box regression loss. The classification loss is calculated *via* binary cross entropy (BCE) and the bounding box regression loss is calculated by a novel metric CIoU_Loss_ which takes overlapping area, center distance, and aspect ratio into consideration. The formula of the CIoU_Loss_ loss is defined in Equations (1–3).


(1) $$\begin{array}{c} CIoU_{Loss}=1-\left(IoU-\frac{{{Dist}_{2}}^{2}}{{{Dist}_{C}}^{2}}\;-\;\frac{\nu^{2}}{\left(1-IoU\right)+\nu }\right) \end{array}$$

(2) $$\begin{array}{c} IoU=\frac{O^{gt}\cap O^{p}}{O^{gt}\cup O^{p}} \end{array}$$

(3) $$\begin{array}{c} \nu =\frac{4}{\pi^{2}}{\left(arctan\frac{w^{gt}}{h^{gt}}\;-arctan\frac{w^{p}}{h^{p}}\right)}^{2} \end{array}$$

*IoU* is the intersection over the union between the candidate localization prediction *o*^*p*^ and the ground truth localization *o*^*gt*^ (rectangle). *Dist*_2_ is the center distance between the prediction and the ground truth, and *Dist*_*C*_ is the diagonal distance of the ground truth. In addition, {*w*^*gt*^, *h*^*gt*^} and {*w*^*p*^, *h*^*p*^} are the width and height sizes of the ground truth and the prediction, respectively.

Moreover, the training of a deep neural network model is generally based on the back-propagation strategy which follows the chain rule to iteratively update the learnable parameters of the model. Gradient descent is used to optimize the training process, and specifically, we adapt the stochastic gradient descent optimization which can be formulated as Equations (4–6).


(4) $$\begin{array}{c} g_{k}=\nabla w_{k}\;+\;w_{k}\;*\;wd \end{array}$$

(5) $$\begin{array}{c} v_{k\;+\;1}=v_{k}\;*\;mu\;+\;g_{k} \end{array}$$

(6) $$\begin{array}{c} w_{k\;+\;1}=w_{k}\;-\;v_{k\;+\;1}\;*\;lr \end{array}$$

Here, *w*_*k*_ is a parameter to be estimated by training, and the parameters *wd*, *mu*, and *lr* denote weight decaying, momentum, and learning rate, respectively.

### Evaluation metric

In accordance with our previous work, we also calculate the recall and the precision given by Equations (6, 7) to evaluate the performance of the trained YOLOv5m models on the image data mentioned in the section of “materials and methods—data acquisition.” Here, the terms *TP*, *FN*, and *FP* are the numbers of true positives, false negatives, and false positives, respectively. The recall metric is to reflect the proportion of the actual positives identified correctly and the precision metric is to answer the question: what is the proportion of the correct positive identification? This metric couple is very significant for the quantitative assessment of the false negative rate and the false positive rate. For instance, if there are a total of 100 diatoms for detection, a recall score of 0.95 means only five objects are not recognized. On the other hand, a precision score of 0.95 represents that 5% of all the detected objects are not diatoms.


(7) $$\begin{array}{c} Recall=\frac{TP}{TP\;+\;FN} \end{array}$$

(8) $$\begin{array}{c} Precision=\frac{TP}{TP\;+\;FP} \end{array}$$

The precision-recall curve is another metric to reflect the overall performance of the trained models on a given data. Previously (Yu et al., 2021), we obtained a set of precision-recall measurements by manually setting different confidence threshold values for inference and plotted them as a precision-recall curve. Hereby, we propose a more elaborate definition of the precision-recall curve that is based on the following steps:

1. Sort the confidence scores (i.e., probabilities) of all the diatom candidates outputted from a trained AI model in descending order;
2. Iteratively update the confidence threshold from 0 to 1 with the change of a small step like 0.01. The threshold is the lower limit of accepting a detected object as a diatom according to its confidence score. For each threshold, we can calculate a couple of precision-recall values, and then a group of precision-recall values can be obtained by changing the confidence threshold;
3. Plot these precision-recall points as a curve and optionally smooth them if necessary.

The area under a given precision-recall curve (AUC), also known as average precision (AP), is a metric for assessing the overall accuracy of a model. In general, a higher AUC score indicates potentially better performance on the same test dataset, and ideally a perfect case would be subject to the AUC score of 1.

The prediction on an SEM image *via* the trained YOLOv5m model depends on the inference settings of not only the already-mentioned confidence threshold but also the IoU threshold as the lower boundary of the overlapping level between a diatom candidate and the ground truth to decide whether the candidate can be accepted as a diatom. There are two more evaluation metrics associated with the IoU threshold: AP@0.5 and AP@0.5:0.95 that are involved as part of the measurements in our study. The metric AP@0.5 is the average precision at the IoU threshold of 0.5 and the metric AP@0.5:0.95 is the mean value of all the APs corresponding to the IoU threshold setting from 0.5 to 0.95 with an interval of 0.05.

To evaluate the performance of the multi-class diatom recognition on the lab-grown diatom samples, we introduce another two evaluation methods called mAP and confusion matrix into this study. For each diatom genus, there is an AP@0.5/AP@0.5:0.95 score and the mAP is essential to calculate the mean value of the average precisions in terms of all the classes. Therefore, in the case of the multi-class diatom recognition, we can also achieve the mAP@0.5 and mAP@0.5:0.95 scores other than the previous AP@0.5 and AP@0.5:0.95 for each genus. On the other hand, the confusion matrix in our scenario is a way of observing the implicit correlation among different diatom genera. Specifically, it demonstrates the relations in a matrix where the sum of each row is the actual number of one genus and each column includes the prediction results of each genus for a specific diatom class, in such a way that the number of correct and incorrect predictions are summarized with their counts and are broken down by each class. This can help us to find out which classes are hard to be differentiated and further can guide us to design more reasonable algorithms for distinguishing them. Note that the recall score of each class can be directly computed from the confusion matrix according to the definition of Equation (6).

### Settings

The following studies were conducted on the hardware and software environments summarized in Table 3. All the scanned SEM images have the same image size 1,024 × 1,024 and considering the trade-off between the available computation resource of the Nvidia RTX 2080Ti GPU in Table 3 and a reasonable batch size, we resized each SEM image to either 800 × 800 or 640 × 640 before feeding it into the YOLOv5m model. For these two input image sizes, the corresponding batch sizes are 16 and 28, so that they are not too small and are not prone to cause the oscillation of training. The training parameters *wd*, *mu*, and *lr* for the stochastic gradient descent optimization are set to be 5e-4, 0.937, and 0.01. Moreover, we define the complete pass of a training dataset as an epoch, and the epoch number of every single training is set to be 100 for all the proposed studies. For a single training, each model instance after an epoch was used to test the given test data set, and we denote the model achieving the best AP@0.5 score as Best-640/Best-800 and the finally obtained model after 100 epochs as Last-640/Last-800.

**Table 3:** The configuration of hardware and software environment for evaluation.

Hardware	CPU	Intel Xeon CPU E5-1620 v2 @ 3.70GHz
	RAM	24GB
	GPU	NVIDIA GeForce RTX 2080 Ti (×1)
	Video Memory	12GB
	Hard Disk	500GB
Software	OS	Windows 10
	Programming Toolkit	Python 3.9 + PyTorch 1.9 + CUDA 11.1
	IDE	PyCharm Professional

## Results

### Study on the samples of five lab-grown diatom genera

According to the summary of Table 1, we collected five lab-grown diatom genera that are *Coscinodiscus, Cymbella, Navicula, Nitzschia*, and *Synedra*. There are around 2,000 images prepared for each diatom genus, and about 46% of all the images have at least one diatom. We applied 4-fold cross-validation on the available images to evaluate both the single-class detection of the diatoms and the multi-class diatom recognition. Specifically, we partitioned the images of each genus into two categories depending on the criterion if one image contains at least one diatom or not. Furthermore, we uniformly divided the images of every category into four groups, and then all the images labeled with the same group index were mixed for the 4-fold cross-validation. In each fold, one group was picked for validation, and the rest three groups were used for training. All the models were initialized by the pre-trained parameters learned on the image dataset ImageNet (Girshick et al., 2014) before training.

**Figure 5:**
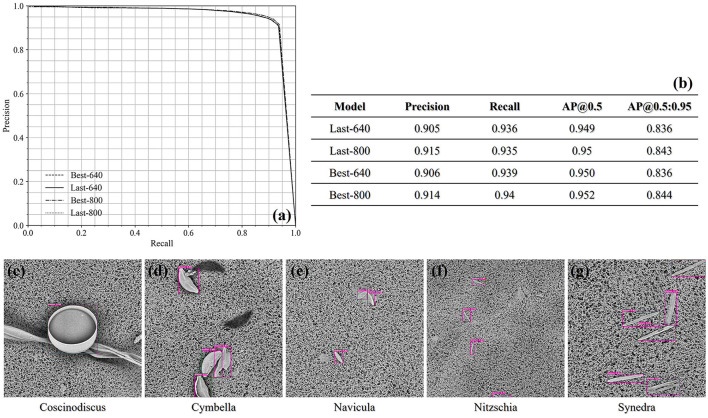
**(a)** The precision-recall curves of the single-class diatom detection under the confidence threshold 0.5 and the IoU threshold 0.5. **(b)** The precisions and recalls at the confidence threshold 0.5 achieved by different models. **(c–g)** The qualitative demonstration of the detection cases of the five test genus.

For the single-class diatom detection, all the results achieved at the confidence threshold of 0.5 are outlined as a table in Figure 5. We can find that the input image size of 800 × 800 is slightly superior to 640 × 640 in terms of precision, and the recall score achieved by the Best-800 model has reached 0.94 while the corresponding precision score is 0.914. Besides, no matter whichever model, the AP@0.5 score is always around 0.95, which demonstrates the capability of the trained YOLOv5m models in handling the standard samples. The precision-recall curves are plotted in the left-upper corner of Figure 5, and we also exhibited the detection cases of all five genera. Here, it is noticeable that the sizes of the diatom genera are quite different, which proves that the YOLOv5m architecture enables to capture the objects on a large scale.

In a forensic diatom test, the recall should be more important than the precision, in that the false positives can be possibly corrected *via* some post-processing strategies, such as an individual classification after the current detection. Since the precision and the recall are commonly a couple of measurements standing by the false negative rate and the false positive rate, respectively, we modulated the confidence threshold and achieved different results. Especially, when the confidence threshold is set to be 0.4, the recall score achieved by the Best-800 model is slightly higher than 0.95, while the associated precision score is 0.9.

**Figure 6:**
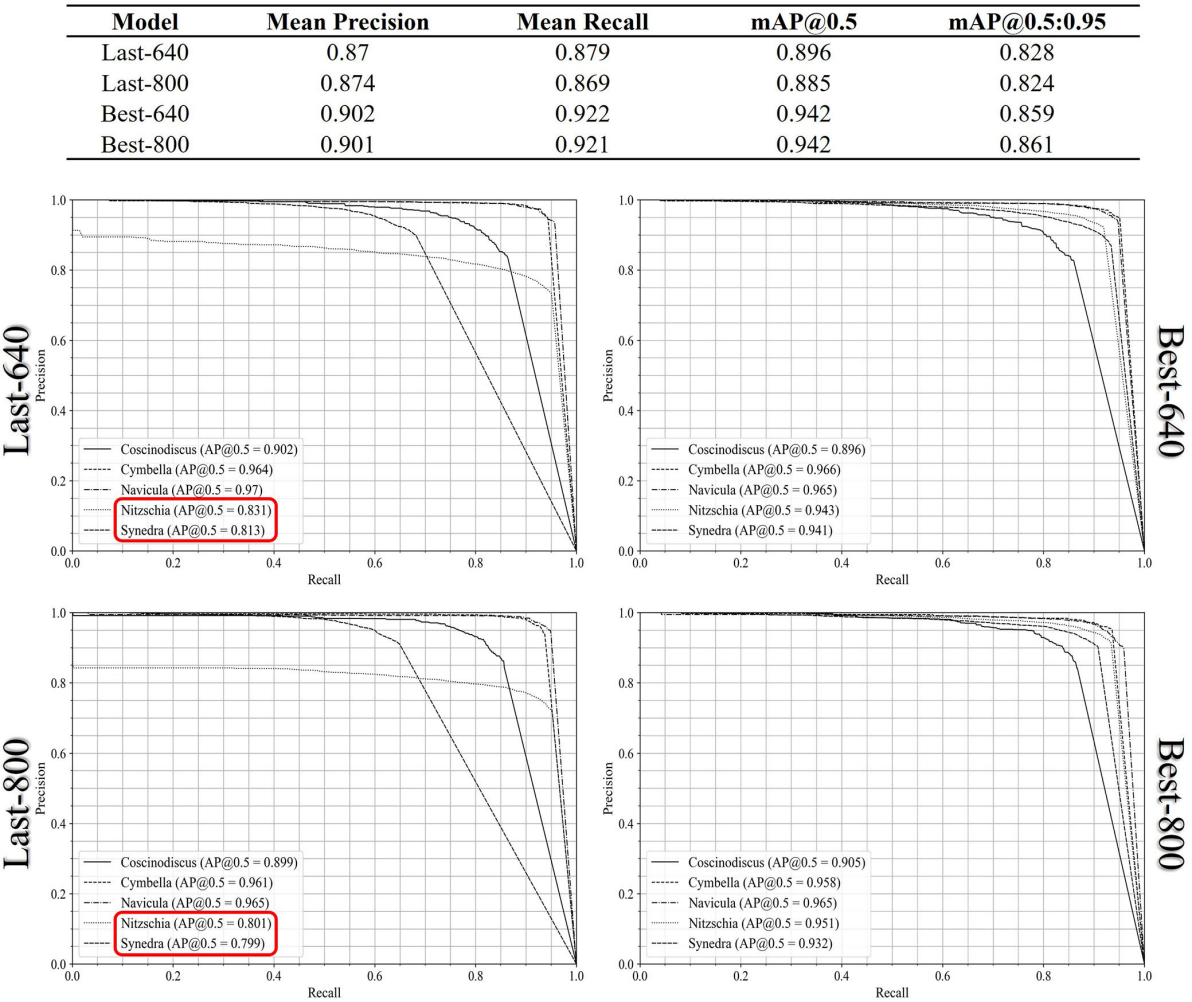
The precision-recall curves of all the diatom genus achieved by the Last-640, Last-800, Best-640, and Best-800 models.

For the multi-class diatom recognition which includes the diatom detection and the classification of every diatom candidate with a genus label, we computed the mAP@0.5 score and mAP@0.5:0.95 score from the AP results of each genus and summarized them in Figure 6. In comparison to the previous single-class diatom detection test, there are no remarkable differences between the Best-640 model and the Best-800 model, while the mean recall of the last-640 model is 1% higher than the one of the last-800 model. To get a perception of the model performance in each genus, we plot the precision-recall curves of the five genera in Figure 6. The AP@0.5 scores of the two diatom genera *Nitzschia* and *Synedra* achieved by the Last-640 model are considerably better than the Last-800 model, leading to the overall AP of the Last-640 model being superior to that of the Last-800 model. In addition, we notice that the performances of the Best-800 model on every genus are similar with a smaller variance of the AP@0.5 scores than that achieved by the Best-640 model.

The normalized confusion matrices in terms of the Last-640 and Best-640 models are summarized in Table 4, where we can find some hidden correlations among different genera. For instance, there is a 45% probability of misrecognizing *Nitzschia* as *Synedra* by the Last-640 model and indeed the two genera look rather similar in shape. Also, the size of the Synedra is very small in our standard samples and this genus is easy to be recognized as background. Moreover, among the false positives, more than 80% of them are identified as *Nitzschia* obtained by the Best-640 model. As well, more than 30% and 65% of the false positives detected by the Best-640 model are regarded as *Nitzschia* and *Synedra* individually. Overall, the confusion matrix is a useful tool to indicate the potential intra-class confusion for solution improvement.

**Table 4:** The confusion matrices derived from the multi-class recognition of the lab-grown diatoms with the Last-640 and Best-640 models.

**Last-640**		**Predicted**					
		***Coscinodiscus***	***Cymbella***	***Navicula***	***Nitzschia***	***Synedra***	**Background**
Actual	*Coscinodiscus*	0.94	0	0	0	0	0.06
	*Cymbella*	0	1	0	0	0	0
	*Navicula*	0	0	0.94	0	0.01	0.05
	*Nitzschia*	0	0	0	0.51	0.45	0.04
	*Synedra*	0	0	0	0	0.71	0.29
	Background	0.01	0.01	0.02	0.81	0.15	0
**Best-640**		**Predicted**					
		***Coscinodiscus***	***Cymbella***	***Navicula***	***Nitzschia***	***Synedra***	**Background**
Actual	*Coscinodiscus*	0.94	0	0	0	0	0.06
	*Cymbella*	0	1	0	0	0	0
	*Navicula*	0	0	0.94	0	0	0.06
	*Nitzschia*	0	0	0	0.88	0	0.12
	*Synedra*	0	0	0	0.05	0.88	0.07
	Background	0.01	0.02	0.01	0.31	0.66	0

The boxed values indicate high mis-recognition cases among some diatom genera and background.

### Study on the samples of lung tissue

We applied the evaluation to the lung samples from the drowning cases. Since there are many sediments in the lung of a drowning corpse, it is very challenging to effectively distinguish the diatoms and the non-relevant objects in the test images. Therefore, this study can be regarded as a performance evaluation on the worst cases of the drowning forensic diatom test.

Again, we evenly split the SEM images scanned on the given lung samples into four groups and conducted a cross-validation. As already mentioned, due to the existence of two interference labels “debris” and “other,” as well as the count imbalance among different genera, it is not suitable to launch a multi-class diatom recognition study, instead, we only focus on the search of all the diatoms in the images. Following the denotation of the models trained on the samples of the lab-grown diatom genera, we also compared the results achieved by the Last-640, Best-640, Last-800, and Best-800 models. Note that each training of the cross-validation begins with an initialization by the YOLOv5m model pre-trained on the SEM images of the lab-grown diatom samples, which already have learned some general features of diatoms.

**Figure 7:**
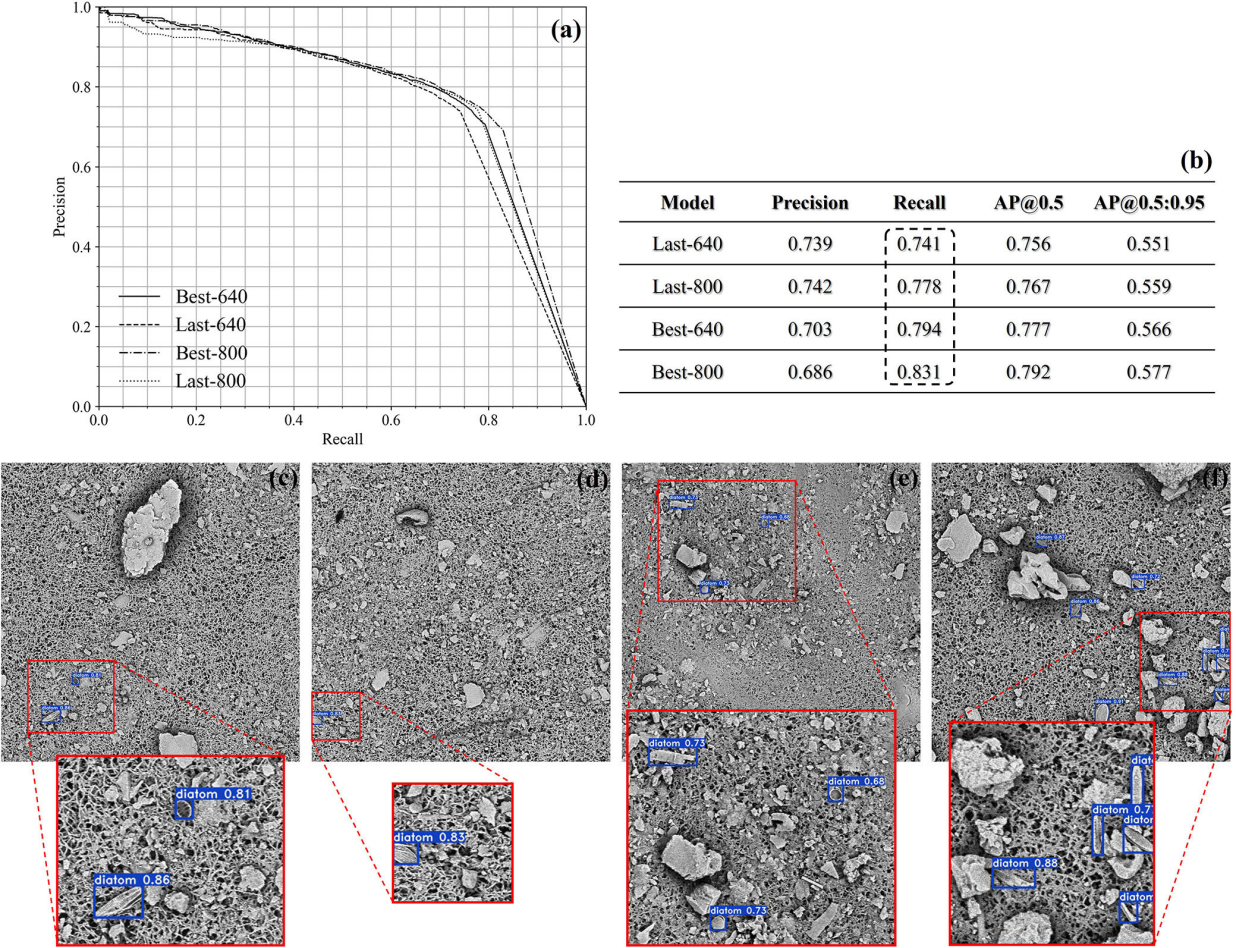
The demonstration of the quantitative results of the lung samples **(a,b)**. Several qualitative cases achieved by the Best-800 model **(c–f)**.

In Figures 7a,b, the precision-recall curves are plotted in the left corner, and in the right corner, there is a summary of the achieved results at the confidence threshold of 0.5. In accordance with the summary, the recall score is getting higher by increasing the size of the images fed into the YOLOv5m from 640 × 640 to 800 × 800. As a result, the Last-800/Best-800 models perform better than the corresponding Last-640/Best-640 models because the textural and morphological information is more abundant. Quantitatively, the best recall score is above 0.8 by the Best-800 model and the corresponding AP@0.5 score is also closed to 0.8. In Figures 7c–f, we qualitatively illustrated several detection cases achieved by the Best-800 model and imposed the confidence score of every diatom candidate on the test images.

### Study on the samples of liver and kidney tissues

**Figure 8:**
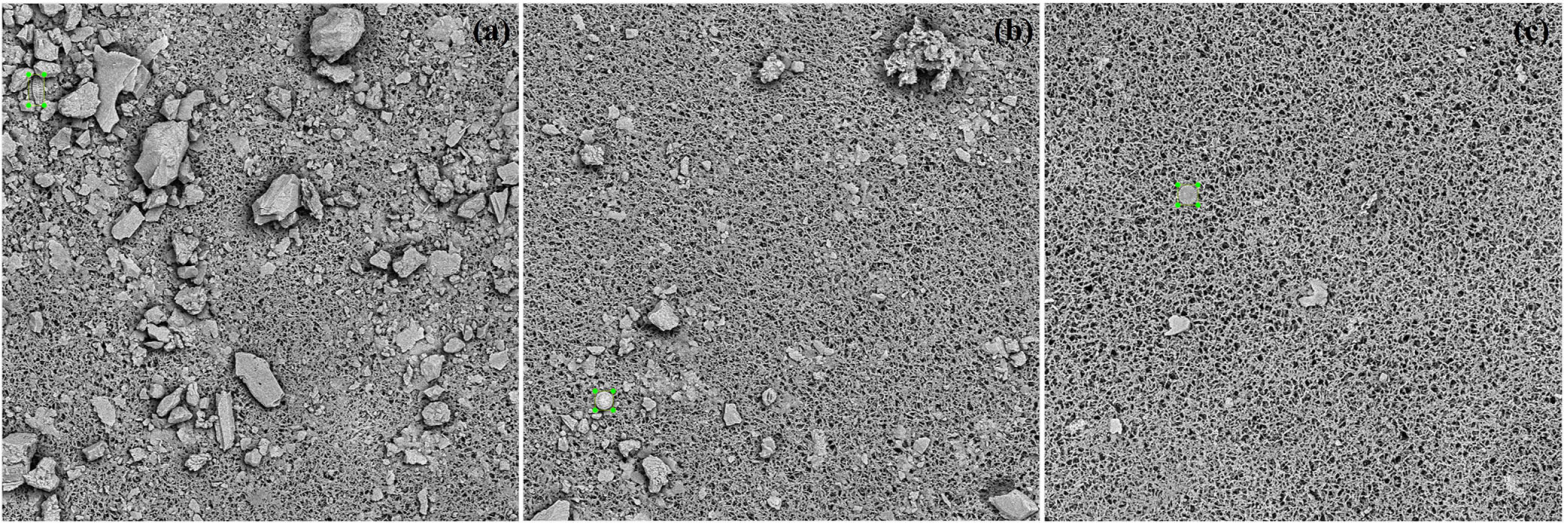
**(a–c)** Three annotated images acquired from the liver and kidney samples with different situations on background.

In this study, we evaluated the image data scanned from the liver and kidney tissues of some drowning corpses. Once more, the available SEM images cannot meet the requirement for a multi-class diatom recognition study due to the already mentioned reasons of the uneven count distribution of each genus, as well as a large portion of diatoms labeled as “other” and “debris.” Hence, we took an evaluation of the single-class diatom detection with a 4-fold cross-validation, while only the input image size 800 × 800 is taken into consideration this time. We initialized each training of the cross-validation with the weights pre-trained on the dataset of ImageNet (Girshick et al., 2014) to reduce the influence of transfer learning. Note that the image dataset used in this study is composed of 11 samples with an obvious variation in the dirty level of the image background which can be found in Figure 8. The image quality of some samples is as poor as that of the previous lung samples, while in the best cases, there are only the diatoms left after the MD-VF pre-processing steps, therefore this study takes the general situation into account for a fair evaluation of the simulation of routine cases.

**Figure 9:**
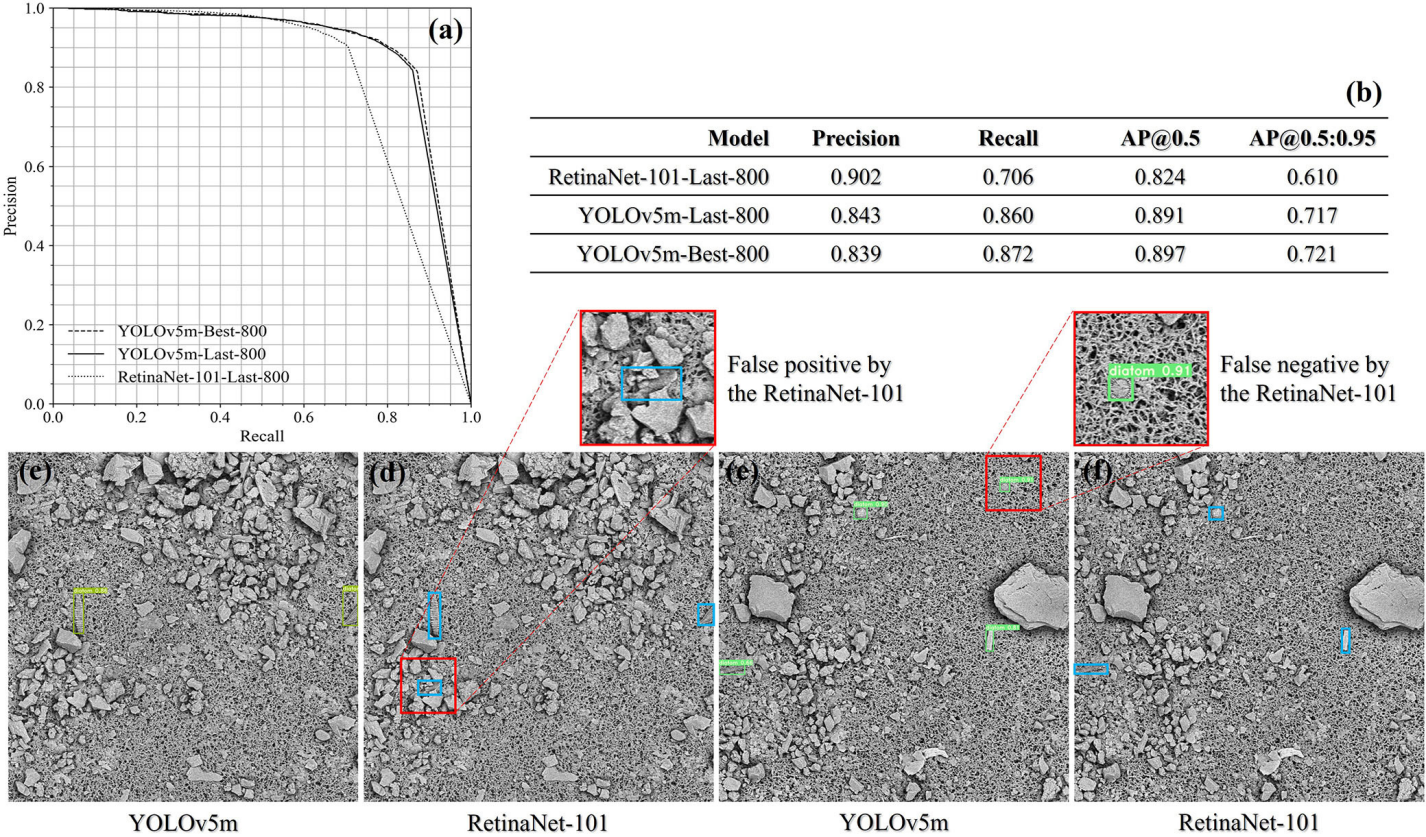
The evaluation result summary of the liver and kidney samples **(a,b)** and two drowning cases qualitatively compared between the YOLOv5m-Last-800 model and the RetinaNet-101-Last-800 model **(c–f)**.

The assessment was conducted on the prepared image data and all the quantitative results are shown in Figures 9a,b. In comparison to the RetinaNet-101 architecture, the YOLOv5m achieved a balance between the precision score of 0.84 and the recall score above 0.86 at the confidence threshold of 0.5. For the same threshold, the RetinaNet-101-Last-800 model is tilted to the precision side, while the false negative rate is therefore much higher than that achieved from the YOLOv5m model. In Figures 9c–f, there are two couples of the diatom detection results predicted by the YOLOv5m-Last-800 model and the RetinaNet-101-Last-800 model.

Since the diatom candidates predicted from a YOLOv5m/RetinaNet-101 model will be filtered both by the IoU threshold and by the confidence threshold defined empirically, we would like to explore the impact of the two threshold parameters on both the precision and the recall to guide our practice. In detail, we kept one threshold at 0.5 and changed the other threshold from 0.1 to 0.5 with a step of 0.1 to observe the trend of performance. All the precision and recall scores are outlined in Tables 5, 6 where the precision and recall scores maintain stable when the IoU threshold is ≤0.5, and when the threshold is above 0.5, the average precision drops down according to the AP@0.5 score and the AP@0.5:0.95 score from the same model as shown in Figure 9. The invariant property of the precision and recall scores shows that most of the true positives and the corresponding ground truth are well overlapped with each other. On the other hand, when progressively changing the confidence threshold from 0.1 to 0.5, the precision score increases while the corresponding recall score decreases, and we notice that the precision and the recall get closed at a low confidence threshold of 0.1 for the RetinaNet-101-Last-800 model, while the YOLOv5m-Last-800 model approaches the balance at the threshold 0.5, which is preferable for practice. In conclusion, the results in Tables 5, 6 indicate that the training in distinguishing the diatoms from the other sediments in the images is much more challenging than learning of predicting the diatom locations.

**Table 5:** The precision and recall scores when changing the IoU confidence threshold from 0.1 to 0.5 and the confidence score is always 0.5.

**IoU threshold**	**YOLOv5m-Last-800**		**RetinaNet-101-Last-800**	
	**Precision**	**Recall**	**Precision**	**Recall**
0.1	0.843	0.858	0.905	0.705
0.2	0.843	0.859	0.905	0.705
0.3	0.843	0.860	0.905	0.705
0.4	0.843	0.860	0.905	0.706
0.5	0.843	0.860	0.902	0.706

**Table 6:** The precision and recall scores by changing the confidence threshold from 0.1 to 0.5 while the IoU score is fixed at 0.5.

**Confidence threshold**	**YOLOv5m-Last-800**		**RetinaNet-101-Last-800**	
	**Precision**	**Recall**	**Precision**	**Recall**
0.1	0.707	0.914	0.778	0.800
0.2	0.764	0.899	0.847	0.764
0.3	0.796	0.887	0.874	0.737
0.4	0.819	0.875	0.891	0.723
0.5	0.843	0.860	0.902	0.706

## Discussion

In this article, we proposed an AI solution to assist the diatom test for searching drowning forensic evidence. Inspired by the huge success of deep learning in various domains (LeCun et al., 2015), we collected the samples from different sources and generated a large image dataset with the microwave digestion and vacuum filtration pre-processing steps (Zhao et al., 2013, 2017) and the image acquisition by a desktop scanning electron microscopy to train our deep learning-based diatom detection models. We adapted the YOLOv5 which is an engineering-optimized version of a well-known object detection architecture (Redmon et al., 2016). If the image data used for training is accompanied by the bounding box annotation, as well as the genus label for every diatom, we can train the multi-class diatom recognition model to predict not only the location of a diatom candidate but also its most possible genus.

As discussed in the “materials and methods—data acquisition” section, the collected samples include three groups for different evaluation purposes *via* cross-validation. All the scanned images have the same size 1,024 × 1,024 and almost all of them were acquired at 1,500× magnification except a liver sample (800× magnification) inherited from our previous work (Yu et al., 2021). For the lab-grown samples of the five specific diatom genera, we evaluated the capabilities of both single-class diatom detection and multi-class diatom recognition. For the former, we tried to achieve the upper limit of the YOLOv5m model considering that the images in this group suffer less from the pollution of impurities. As a result, a recall score of 0.95 together with a precision score of 0.9 are achieved by setting the IoU threshold at 0.5, the confidence threshold at 0.4, and the AP@0.5 score around 0.95. For the latter, it is more challenging due to the extra diatom taxonomy. In conformity with the results reported in the last section, we achieved the best recall score of about 0.92 when the corresponding precision score is 0.9. The difficulty in recognizing the genus of each diatom is not the same, and we observed that the *Nitzschia* and *Synedra* are easy to be misidentified with each other while almost all the false positives are from these two genera.

Both the lung samples and the samples of the liver and kidney tissues were extracted from the drowning cases. We conducted experiments on these samples to estimate the performance of our AI solution in the general situations encountered in the drowning forensic routine. Some interference labels and the distribution of the diatom genera from both groups make the multi-class diatom recognition evaluation not applicable, we therefore care only about the diatom detection issue. Especially, the experiments on the lung samples are designed to evaluate the worst cases due to the existence of various sediments, which indicates the lower limit of accuracy we can potentially achieve in those real cases. In the controlled study of resizing the original image to 800 × 800 and 640 × 640, respectively, as input for training and testing, the best recall score is above 0.83 at the confidence threshold of 0.5 and the corresponding precision score is around 0.7 when the input size is 800 × 800. Also, the AP@0.5 score can reach 0.8. The precision score of 0.7 indicates that there are many false positives that are common for the cases of lung samples, and the candidates predicted from the current model can be further refined by an individual AI model.

On the other hand, we tested the performance of the trained YOLOv5m models on the given liver and kidney images. In comparison to the RetinaNet-101 architecture (Lin et al., 2017) adopted in our previous work, we conducted a 4-fold cross-validation for both frameworks under the same threshold settings. When the confidence threshold and the IoU threshold are both 0.5, the precision score achieved by the RetinaNet-101 (~0.9) is higher than the score from the YOLOv5m (~0.84), while the recall score achieved by the RetinaNet-101 (~0.71) is much lower than the score from the YOLOv5m (~0.87), and the AP@0.5 score of the YOLOv5m can be almost 0.9. Moreover, a balance between the precision score and the recall score was achieved at the confidence threshold of 0.5 and 0.1 corresponding to the YOLOv5m and the RetinaNet-101, which demonstrates the superiority of the YOLOv5m since the RetinaNet-101 is prone to be tilted to the precision side. Besides the mentioned experiments, we also trained a YOLOv5m model using all the images from the liver and kidney samples and deployed it in application software for forensic practice.

In future, we will aim at the completion of our diatom detection and recognition solution by integrating the function of multi-class diatom recognition into it. Since an even distribution of the diatom genera is a prerequisite for training, the annotation on the newly scanned images from more samples is required while there is another possible way for the same purpose by generating many synthetic training images. The multi-class diatom recognition function can be built on either an end-to-end method or a hierarchical strategy, which has been on schedule to be explored.

## Data availability statement

The raw data supporting the conclusions of this article will be made available by the authors, without undue reservation.

## Author contributions

WY and QXi presented concepts, methods and models, wrote the original draft, and wrote and edited the revised manuscript. YH conducted data curation, implemented computer code, and supported algorithms. YD, XK, DZ, HS, QXu, and ZL conducted the experiment and analyzed the data. CL, JZ, and YN offered resources, supervised, and wrote-reviewed and edited the article. All authors read and approved the final manuscript.

## Funding

This study was financially supported by the Grant-in Aids for Scientific Research from the Ministry of Public Security of the People's Republic of China (2020GABJC38, CL), and a grant from the Guangzhou Municipal Science and Technology Project, 2019030001, JZ. Guangzhou Municipal Science and Technology Project, 2019030011, ZL. Guangzhou Municipal Science and Technology Project, 2019030012, CL.

## Conflict of interest

Author WY is employed by Jiangsu JITRI Sioux Technologies Co., Ltd. Author YH was employed by LabWorld (Suzhou) Intelligent Technology Co., Ltd. The remaining authors declare that the research was conducted in the absence of any commercial or financial relationships that could be construed as a potential conflict of interest. The reviewer DL declared a shared affiliation with one of the author YD to the handling editor.

## Publisher's note

All claims expressed in this article are solely those of the authors and do not necessarily represent those of their affiliated organizations, or those of the publisher, the editors and the reviewers. Any product that may be evaluated in this article, or claim that may be made by its manufacturer, is not guaranteed or endorsed by the publisher.

## Footnotes

^1^ https://github.com/ultralytics/yolov5
